# Y-Box Binding Protein 1: Unraveling the Multifaceted Role in Cancer Development and Therapeutic Potential

**DOI:** 10.3390/ijms25020717

**Published:** 2024-01-05

**Authors:** Ngoc Thi Minh Dinh, Tuan Minh Nguyen, Mi Kyung Park, Chang Hoon Lee

**Affiliations:** 1 College of Pharmacy, Dongguk University, Goyang 10326, Republic of Korea; minhngoc@dgu.ac.kr (N.T.M.D.); tuank67a5@gmail.com (T.M.N.); 2 Department of BioHealthcare, Hwasung Medi-Science University, Hwaseong-si 18274, Republic of Korea

**Keywords:** Y-box binding protein 1, YBX1, hallmarks of cancer, neural input, tumor microenvironment, anticancer

## Abstract

Y-box binding protein 1 (YBX1), a member of the Cold Shock Domain protein family, is overexpressed in various human cancers and is recognized as an oncogenic gene associated with poor prognosis. YBX1’s functional diversity arises from its capacity to interact with a broad range of DNA and RNA molecules, implicating its involvement in diverse cellular processes. Independent investigations have unveiled specific facets of YBX1’s contribution to cancer development. This comprehensive review elucidates YBX1’s multifaceted role in cancer across cancer hallmarks, both in cancer cell itself and the tumor microenvironment. Based on this, we proposed YBX1 as a potential target for cancer treatment. Notably, ongoing clinical trials addressing YBX1 as a target in breast cancer and lung cancer have showcased its promise for cancer therapy. The ramp up in in vitro research on targeting YBX1 compounds also underscores its growing appeal. Moreover, the emerging role of YBX1 as a neural input is also proposed where the high level of YBX1 was strongly associated with nerve cancer and neurodegenerative diseases. This review also summarized the up-to-date advanced research on the involvement of YBX1 in pancreatic cancer.

## 1. Introduction

In the year 2020, cancer was responsible for 18% of total deaths, maintaining its position as the second most common cause of death following heart diseases. Pancreatic cancer is the third leading cause of cancer death and reigns supreme in regard of having the lowest 5-year relative survival rates among cancer types [1]. Understanding the mechanism driving tumor progression will be beneficial to cancer treatment. This review aims to offer a comprehensive understanding of Y-box binding protein 1 (YBX1)’s multifaceted role in cancer by summarizing its significant functions in relation to cancer hallmarks [2,3,4].

YBX1 is consistently expressed in various human cancers, including pancreatic cancer [5], breast cancer [6,7], lung cancer [8,9], multiple myeloma [10], osteosarcoma [11], synovial sarcoma [12], prostate cancer [13], and ovarian cancer [14]. It is recognized as an oncogenic gene, frequently overexpressed, and is often associated with a poor prognosis.

By elucidating the intricate mechanisms through which YBX1 influences various facets of cancer biology, we propose its potential as both a prognostic marker and a therapeutic target. Furthermore, the role of YBX1 in neurogenesis is also discussed, emphasizing its impact on the tumor microenvironment. Additionally, we discuss current trends and strategies in the development of anticancer drugs targeting YBX1. Several clinical trials have commenced addressing YBX1 for cancer treatment, and the burgeoning preclinical research on YBX1 inhibitors underscores its clinically significant implications. The growing interest in YBX1 in pancreatic cancer will also be discussed.

## 2. YBX1: Multifaceted Roles in Biology

The various functions of YBX1 are well presented in recent papers, so please refer to them. Here, we will only briefly introduce the most basic functions. YBX1 is a versatile protein belonging to the highly conserved Cold Shock Domain family [15,16]. This 36 kDa protein comprises an N-terminal domain rich in alanine and proline, a Cold Shock Domain, a domain believed to function as an acidic nucleotide binding site, and a C-terminal domain characterized by alternating clusters of positively and negatively charged amino acid residue [17].

YBX1 demonstrates an ability to bind to a wide array of DNA and RNA molecules, implicating it in numerous cellular functions. Initially identified as a transcription factor, YBX1 specifically binds to a DNA sequence called the Y-box (5′CCAAT3′) found in the promoter and enhancer regions of many genes, implying its involvement in regulating various gene expressions [18,19,20].

Over time, additional roles of YBX1 have been uncovered, including its function as an RNA-binding partner and its participation in processes such as splicing, translation, mRNA stability, and RNA sorting. These processes are frequently associated with RNA processing within exosomes. Moreover, independent studies have shed light on various other specific aspects of YBX1’s involvement in cancer development.

## 3. Effects of YBX1 on Cancer Hallmarks Originated from Cancer Itself

### 3.1. Role of YBX1 in Sustaining Proliferative Signaling and Evading Growth Suppressors

Uncontrolled proliferation resulting from dysregulated growth is a notable characteristic observed in cancer cells. Overexpression of YBX1 facilitates the growth of pancreatic ductal adenocarcinoma (PDAC) through the GSK3B (glycogen synthase kinase 3 beta)/cyclin D1/cyclin E1 pathway [21]. In pancreatic tubular adenocarcinoma, dual luciferase reporter and ChIP experiments have demonstrated that YBX1 binds to the promoter of GSK3B, thereby promoting its transcription and leading to elevated levels of GSK3B protein. This, in turn, increases the expression of Cyclin D1 and E1, facilitating the transition of the cell cycle from the G1 phase to the S phase [21]. Nuclear accumulation of YBX1 has been found to exhibit a positive correlation with increased levels of Cyclin A and Cyclin B1 mRNA and protein [22]. Aberrant expression of YBX1 is linked to adhesion-independent cell proliferation, primarily through the induction of Cyclin A [22]. The upregulated long non-coding RNA (lncRNA) LINC00665 in gastric cancer interacts with YBX1, activating Wnt/β-catenin signaling, promoting tumor progression [23].

YBX1 overexpression also induces constitutive activation of the epidermal growth factor receptor (EGFR), which contributes to the proliferation of breast cancer cells [24]. Moreover, YBX1 enhanced *EGFR* transcription by directly binding to its promoter in chordoma cells, thereby regulating protein expression of p-EGFR, p-AKT and its downstream target genes that influence cell apoptosis and cell cycle transition [25]. Overexpression of YBX1 in breast cancer induces EGFR expression and vice versa, in which the pivotal role of S102 is proved in YBX1 binding EGFR promoter capability [6,26]. The EGFR proteins are actively involved in cell signaling pathways that oversee processes like cell division and survival. This implies that when YBX1 is induced to activate the EGFR signaling pathway, it can potentially influence and adjust cell proliferation and the ability of cells to survive. Intriguingly, the activation of YBX1 within the cell nucleus is facilitated by the crucial cell growth signal known as the PI3K/AKT pathway [6,14,27]. This creates a feedback loop that further intensifies cell proliferation.

YBX1 collaborates with oncogenic lncRNA SBF2-AS1, driving breast cancer cell proliferation via PI3K/AKT/mTOR [28]. DARS1 Antisense RNA 1 interacts with YBX1, leading to enhanced binding and stabilization of target mRNA [29]. This interaction establishes a combined transcriptional and post-transcriptional feed-forward loop that upregulates the expression of critical regulators involved in the G1-S transition, such as E2F1 and Cyclin D1. Furthermore, DARS1 Antisense RNA 1/YBX1 also contributes to the stabilization of Forkhead Box M1 mRNA, a master transcription factor responsible for governing glioblastoma (GB) stem cell self-renewal and DNA double-strand break repair. RP11-162G10.5, a histone-driven lncRNA in luminal breast cancer, worsens outcomes when overexpressed. It spurs tumor growth by involving YBX1, activating GLO1, spotlighting YBX1’s critical role in breast cancer advancement [30].

Post-transcriptional modification of YBX1 plays a role in regulating YBX1 location and functions. The most well-researched PMT of YBX1 is the phosphorylation at S102 by AKT and the Ribosomal Protein S6 Kinase A1 (p90RSK) family which facilitates nuclear translocation, leading to the enhancement of cell proliferation and survival [27,31]. In response to everolimus (mTORC1 inhibitor) or TAS0612 (multikinase inhibitor of AKT, p70S6K, and p90RSK), YBX1 phosphorylation at S102 was downregulated together with the inhibited cell growth and abrogated antiestrogen resistance in breast cancer cells [32]. Additionally, it is noteworthy that the phosphorylation of YBX1 at other specific sites such as S176 and S165 and R205 methylation play a crucial role in activating NF-κB which is involved in driving cell proliferation in colon cancer [33,34,35].

Besides acting as an oncogene, some studies also implied that YBX1 can be secreted and extracellular YBX1 can act as a extracellular mitogen and chemoattractant that orchestrates cell migration and proliferation [36,37].

Although certain studies have indicated that elevated levels of YBX1 can enhance cancer cell growth in GBM [38] and breast cancer cell lines [39], contrasting evidence suggests that enforced YBX1 expression actually suppresses cell proliferation. This suppression occurs through the block of cap-dependent translation of growth-related mRNAs, while simultaneously enhancing mesenchymal genes expression specifically in breast cancers [40,41]. YBX1-overexpressing cells display a remarkable ability to shut down proliferation and survive in anchorage-independent conditions [41]. These results suggest an intriguing hypothesis that YBX1 may play a role in inhibiting cap-dependent translation of growth-related mRNAs, while simultaneously activating Internal Ribosome Entry Site (IRES)-dependent translation of the mesenchymal markers genes in tumor regions characterized by hypoxia and nutrient deprivation. This dual mechanism potentially promotes the acquisition of mesenchymal-like migratory properties and facilitates the spread of the tumor.

In a separate study focusing on liver cancer, researchers observed that reducing YBX1 expression significantly decreased cell proliferation in MHCC97H and HCCLM3 cells, while overexpressing YBX1 in BEL7402 and SMMC7721 cells inhibited cell growth [42]. These discoveries support the idea that although increased proliferation is crucial for the initiation and sustenance of primary tumors, growth inhibition may be vital for the survival of cancer cells within the bloodstream and secondary organs, ultimately leading to the emergence of a more aggressive cancer phenotype.

During the G1 phase of the cell cycle, the Cyclin/CDK complex plays a pivotal role in hyperphosphorylating RB, leading to the liberation of transcription factors known as E2Fs from RB inhibition [43]. These released E2Fs subsequently activate the transcription of multiple genes that facilitate the progression of the cell cycle from G1 to the S phase [44]. It is worth noting that inhibiting YBX1 through knockdown has been observed to reduce the levels of Cyclin D1 protein in multiple myeloma cells [10,45]. In pancreatic adenocarcinoma cells, the overexpression of YB1 increased the expression of Cyclin D1 and E1 [21]. Furthermore, findings from reporter luciferase experiments and chromatin immunoprecipitation (ChIP) studies have indicated that YBX1 binds to the Cyclin D1 promoter, enhancing its expression at both the protein and mRNA levels in lung cancer cells and neuroblastoma cells [46,47]. These results suggest that YBX1, functioning as an upstream regulator of RB, plays a critical role in initiating the cell cycle.

CDC25a (Cell division cycle 25A) serves as a vital regulator of the cell division cycle and plays a central role in facilitating the progression of the cell cycle by removing inhibitory phosphorylation on proteins such as CDK2 (Cyclin Dependent Kinase 2), CDK4, and CDK6 [48,49,50,51]. The binding of YBX1 to the CDC25a promoter region was confirmed through ChIP and luciferase experiments. Elevated levels of both YBX1 and CDC25a proteins were observed in lung adenocarcinoma cells compared to lung fibroblasts. Conversely, when YBX1 is inhibited in A549 and H322 cells, its binding capacity to the CDC25a promoter significantly decreases. This leads to cell cycle arrest, the inhibition of cell proliferation, and the induction of apoptosis [52]. Additionally, inhibiting YBX1 results in a significant reduction in the S phase content, along with a notable decrease in CDC6 (Cell Division Cycle 6) expression in breast cancer cells [45] and chordoma cells [25].

Inhibition of YBX1 by knockdown results in decreased expression of the cell cycle “activator” E2F2 (E2F transcription factor 2) and an increase in the expression of the “repressor” E2F5. Moreover, ChIP experiments have revealed that endogenous YBX1 binds to the promoters of classical E2F1-regulated genes in MCF-7 cells, suggesting that YBX1 may act as a potential transcriptional regulator of these genes [53]. Another study has confirmed that the promoters of E2F1, E2F2, E2F3, E2F4, and E2F7 are targeted by YBX1 [54].

YBX1, vital in cell survival for solid tumors and acute myeloid leukemia, shows heightened levels in T cell acute lymphoblastic leukemia (T-ALL). Its depletion impedes T-ALL progression by reducing proliferation, increasing apoptosis, and altering key signaling pathways [55].

YBX1 plays a crucial role in pleural mesothelioma (PM) growth and migration; genetic knockdown or entinostat targeting YBX1 significantly inhibits PM cell growth and enhances sensitivity to cisplatin and radiation, particularly through increased platinum uptake [56]. Combining entinostat with cisplatin shows strong synergistic effects, suggesting YBX1 as a potential target for enhancing treatment responses in PM.

**Figure 1:**
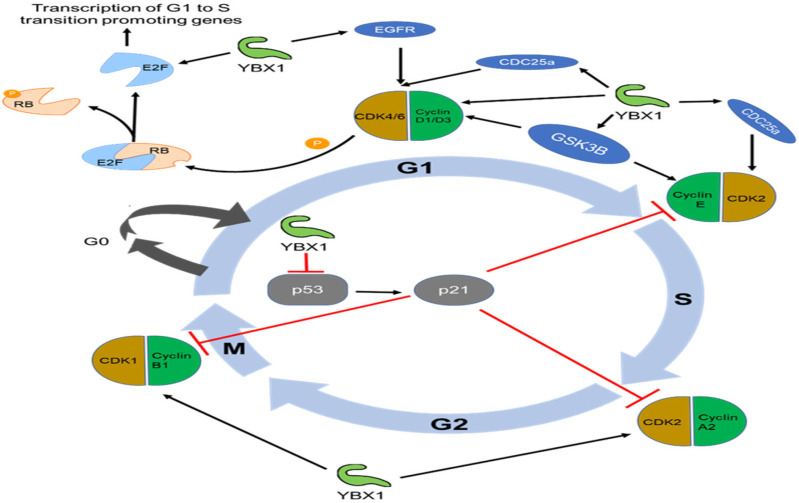
YBX1 plays a crucial role in cell cycle regulation. YBX1 transcriptionally increases the expression of E2F, Cyclin D1, CDC25a, GSK3B, and EGFR, thereby facilitating cell cycle activation and progression. The expression of YBX1 is positively correlated with various cell cycle-related proteins that contribute to heightened cell proliferation. Abbreviations: YBX1: Y-box binding protein 1; CDC25a: cell division cycle 25A; GSK3B: glycogen synthase kinase 3 beta; EGFR: epidermal growth factor receptor.

In summary, YBX1 has the capacity to control the expression of a wide range of cell cycle-related genes, encompassing both “activators” and “repressors”. This suggests its role as a pivotal “cell cycle switch” that coordinates and directs the progression of the cell cycle, as depicted in Figure 1. 

### 3.2. Role of YBX1 in Cell Death Resistance

To comprehend the role of ybx1 in resistance to cell death, it is essential to first grasp the concept of cell death itself. Cell death comes in various forms, with apoptosis and necrosis being two broad categories [57].

In particular, apoptosis involves a complex machinery comprising both upstream regulators and downstream executioners [58,59]. The regulators can be categorized into two primary circuits: The first circuit is responsible for receiving and processing death-inducing signals originating from the external environment. These signals are known as extrinsic apoptotic programs and include events like the interaction between Fas ligands and Fas receptors. The second circuit is tasked with sensing and integrating various signals arising from within the cell itself, referred to as the intrinsic apoptotic program. Both of these circuits ultimately culminate in the activation of caspases 8 and 9, which in turn activate executioner caspases responsible for carrying out the apoptotic process.

**Figure 2:**
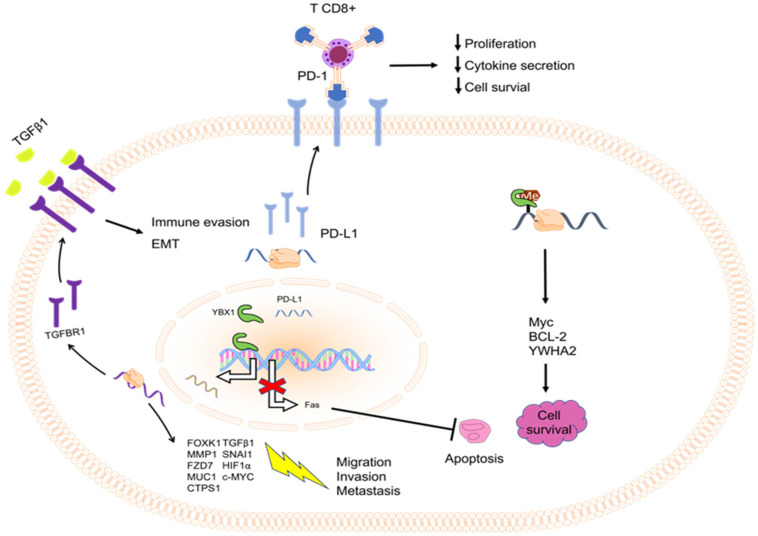
Role of YBX1 in cell death decision, migration and invasion activation, and immune destruction evasion. YBX1 transcriptionally and translationally regulates the expression of a vast range of genes, which eventually attenuate cell survival, the epithelial–mesenchymal transition process, and avoid+ immune surveillance.

YBX1 functions as an inhibitor of Fas-related cell death by being part of a complex that binds to the repressor region of the Fas promoter [60]. Knockdown of YBX1 is suggested to be related to apoptosis induction in myeloid leukemic cells [61,62], renal cell carcinoma [63], colon cancer [64], medulloblastomas [65], glioma cells [66], prostate cancer [67], and lung adenocarcinoma [52]. However, its role in apoptosis in pancreatic cancer has not yet been reported [68,69]. In myeloid leukemia, YBX1 interacts with insulin-like growth factor 2 messenger RNA binding proteins and promotes cell survival by stabilizing them through the binding of m6A as a target, specifically to Myc, BCL2, and YWHAZ mRNAs [62,70], as described in Figure 2. Remarkably, the loss of YBX1 does not seem to have any discernible impact on normal hematopoiesis [62]. Therefore, the selective role of YBX1 in cancer suggests that it may be a promising target for more comprehensive research and treatment of myeloid leukemia.

Moreover, Myc binds to the promoter of the MNX1 Antisense RNA 1, which is a lncRNA whose expression is de-repressed in colon cancer and activates its transcription. MNX1 Antisense RNA 1 can bind to YBX1, preventing its ubiquitination and degradation [71]. This implies the existence of a potential reciprocal regulatory loop between Myc and YBX1, which could play a role in maintaining antiapoptotic signaling in tumors. In vincristine-resistant rhabdomyosarcoma (RMS) cells, heightened CD133-positive stem-like cells revealed MYC and YBX1 as critical transcription factors [72]. Targeting the MYC-YBX1 axis reduced stem-like traits, suggesting YBX1’s pivotal role and potential as a therapeutic target in RMS treatment.

The circular RNA (circRNA) circRABL2B negatively correlates with MUC5AC in lung cancer, and patients with low circRABL2B and high MUC5AC exhibit poorer survival. Notably, circRABL2B interacts with YBX1 to inhibit MUC5AC, suppressing integrin β4/pSrc/p53 signaling, reducing cell stemness, and enhancing erlotinib sensitivity, highlighting YBX1 as a key player in circRABL2B-mediated antineoplastic effects [73].

Furthermore, post-transcriptional modifications of YBX1 play a role in regulating apoptosis. Polo-like kinase 1, a member of the serine/threonine protein kinase family, directly phosphorylates YBX1 at serine 174 and serine 176. This phosphorylation is crucial for the nuclear translocation of YBX1 and its involvement in apoptosis surveillance in glioma stem cells [74]. CREB1-activated PIN1P1 contributes to gastric cancer progression by interacting with YBX1 and inducing PIN1 upregulation [75]. Oncogenic AURKA influences breast cancer-related RNA splicing, interacting with YBX1 to promote GOLGA4 exon inclusion and with hnRNPK to induce RBM4 exon skipping [76]. The AURKA-YBX1/hnRNPK complex correlates with poor breast cancer prognosis, and blocking nuclear AURKA shows promise in reversing oncogenic splicing events, highlighting YBX1’s significant role in this process.

YBX1 in the nucleus inhibits the apoptosis-inducing ability of p53 and is associated with the failure to increase levels of the Bax protein following stress-induced activation of p53 in normal mammary epithelial cells [77]. In hepatocellular carcinoma (HCC), YBX1 plays a pivotal role, regulating FCN3 to SBDS and participating in a feedback loop involving p53 and SBDS downstream of FCN3 [78].

YBX1 demonstrates the capacity to evade cell death signals, not only in cancer cells but also in immune cells. The expression of YBX1 was significantly lower in apoptotic T cells and activated T cells from Systemic Lupus Erythematosus (SLE) patients compared to nonapoptotic T cells and activated T cells from healthy individuals [79]. Knockdown of YBX1 in T cells resulted in the upregulation of proapoptotic molecules, activation of caspase-3, and subsequent induction of apoptosis. YBX1-mediated survival in T cells was reversed following the inactivation of AKT and phosphoinositide 3-kinase (PI3K). Restoring YBX1 expression in SLE patients significantly improved T cell survival and prevented cell death, even in T cells that are highly susceptible to apoptosis [79].

Unlike apoptosis, which is a process of programmed cell death, necrosis is typically characterized by the swelling and rupture of cells, resulting in inflammation and damage to surrounding tissues [57]. It is often considered an uncontrolled and pathological form of cell death.

It has been reported that the nuclear accumulation of the human transcription factor YBX1 facilitates the viral DNA replication of E1-deleted adenoviral vectors by regulating the adenoviral E2 late promoter [80]. Interestingly, the overexpression of YBX1 in human cells not only increases viral DNA replication but also promotes a necrosis-like cell death, leading to the release of viral particles [81]. Therefore, cancers with high expression of YBX1 provide a potential opportunity for cancer therapeutic intervention using conditionally replicating adenoviruses. This suggests that side effects in normal tissues with low YBX1 expression may be minimized. However, further studies are needed to fully understand the molecular mechanisms responsible for the accelerated cell destruction by YBX1 and to elaborate the potential of adenovirus-based gene therapy for cancer treatment.

### 3.3. Role of YBX1 in Enabling Replicative Immortality and Senescence

Cellular senescence refers to a state in which the cell cycle is permanently arrested and has traditionally been considered a mechanism to suppress tumorigenesis and maintain homeostasis in the body. Various conditions, such as nutrient deficiencies, DNA damage, organelle damage, disruption of cellular structures, and imbalances in cell signaling, can induce senescence in cells [82,83].

YBX1 plays a role in preventing senescence in skin progenitor cells, and a reduction in YBX1 expression induces senescence by binding to the 3′ untranslated region of certain senescence-associated secretory phenotype (SASP) factors, including IL-8 and fibroblast secretory protein (CXCL1), thereby inhibiting their translation. Conditioned media from YBX1-inhibited cells significantly increases the number of human keratinocytes undergoing cell cycle arrest, as indicated by positive Senescence β-galactosidase staining, compared to cells grown in control media [84]. YBX1 is identified as a crucial carrier for loading satellite II (SATII) RNA into small extracellular vesicles (sEVs) [85]. These sEVs containing SATII RNA, regulated by YBX1, induce cellular senescence and enhance the expression of inflammatory SASP genes.

Depletion of YBX1 in immortal keratinocytes restores senescence and disrupts the connection between cell cycle arrest and the SASP [86]. Additionally, overexpression of YBX1 suppresses senescence characteristics by binding to the p16 promoter, thus increasing p16 expression levels [87,88]. Furthermore, the loss of YBX1 in bone marrow stromal cells has been linked to aging and bone loss in both mice and humans [89].

Recent research has revealed that when cells undergo senescence, they release a variety of inflammatory molecules and factors, including inflammatory cytokines, chemokines, growth factors, and matrix remodeling factors (SASP). These substances have the potential to impact the local tissue environment, thereby playing a role in the development of chronic inflammation and the progression of cancer [90,91]. Moreover, it is noteworthy that senescent cells can not only facilitate tumor progression in cancer cells but also affect various cell types within the tumor microenvironment [82,92,93].

YBX1 plays a role in controlling the expression of SASP elements when it is phosphorylated at a specific site, namely S102. Long noncoding RNA MIR31HG interacts with YBX1, which promotes the phosphorylation of YBX1 at serine 102 by the kinase RSK. When YBX1 is more heavily phosphorylated at S102, it leads to higher levels of IL1A protein. This IL1A protein serves as an upstream regulator of SASP components like CXCL1, IL-8, and IL-6, ultimately promoting cell invasion through matrix-coated membranes [94]. Interestingly, MIR31HG exhibits oncogenic properties in PDAC, and its expression is downregulated by miR-193b [95].

YBX1 is associated with the promotion of the expression of genes related to cancer stem cells. When YBX1 is reduced (knocked down) in mouse embryonic stem cells, there is an increase in the mRNA levels of differentiation-related genes like Brachyury, Mix1l, Mef2c, and Isl1 [96]. This suggests that YBX1 plays a role in preserving stem cell characteristics and supporting their continuous proliferation.

Furthermore, ChIP assays have revealed that CD44 is a target gene of YBX1. Elevated levels of the stem cell signature CD44, coupled with the ability to form mammospheres in trastuzumab-resistant breast cancer cells, are regulated by YBX1 phosphorylated at S102. This regulation contributes to the cancer cells’ capacity to resist trastuzumab treatment [97]. Through ChIP-seq analysis, YBX1 is shown to sustain the stemness of cancer stem cells by enhancing the expression of genes associated with stemness, such as FZD1 (Frizzled Homolog 1), p21, GLP-1R (Glucagon Like Peptide 1 Receptor), GINS Complex Subunit 1, and Notch Receptor 2 [98]. Additionally, the downregulation of YBX1 in basal-like breast cancer cell lines MDA-MB-231 and BT549 is strongly correlated with a significant reduction in the mRNA expression levels of cancer stem cell genes, including POU Domain Transcription Factor OCT4, Homeobox Transcription Factor Nanog, and SOX2 (SRY-Box Transcription Factor 2) [99].

### 3.4. Role of YBX1 in Activating Invasion and Metastasis

In metastasis, cancer cells break away from the original (primary) tumor, travel through the bloodstream or lymphatic system, and form new tumors in other organs or tissues of the body [100,101]. Epithelial-mesenchymal transition (EMT) is a potential mechanism through which transformed epithelial cells can acquire invasive capabilities, resist apoptosis, and proliferate [102]. One of the key contributors to this process is TGFβ1 (Transforming Growth Factor Beta 1), a well-known inducer that triggers EMT in various types of epithelial cells [103].

RMRP (RNA Component Of Mitochondrial RNA Processing) enhances the proliferation, invasion, and migration of NSCLC (non-small cell lung cancer) cells in a TGFBR1/SMAD2/SMAD3-dependent manner by recruiting YBX1 to the promoter region of TGFR1(Transforming Growth Factor Beta Receptor 1), thereby increasing the transcription of TGFBR1 [104].

Distinct head and neck cancer subtypes lack specific treatments; integration of single-cell sequencing and proteome profiles reveals an EMT signature linked to PI3K/mTOR activity, proposing YBX1 phosphorylation as a potential therapeutic target to shift mesenchymal cells for treatment [105].

In the context of nasopharyngeal carcinoma, reducing the presence of YBX1 through knockdown had a significant negative impact on cell migration and invasion [106,107]. Moreover, this knockdown also had a partial inhibitory effect on TGFβ1-induced cell migration, and it was associated with changes in markers linked to EMT [108].

In stable YBX1 knockdown breast cancer cells, there was a substantial decrease in both mRNA and protein levels of matrix metalloproteinase-1 (MMP-1) and beta-catenin. These molecules are well-recognized contributors to cancer metastasis. YBX1 was found to function as a transcription factor for MMP-1, playing a role in its regulation [109].

Additional evidence supporting the role of YBX1 in regulating cell migration, invasion, and metastasis has been reported in various contexts. YBX1 expression drives pancreatic cancer metastasis and is counteracted by microRNA(miR)-216a [110]. In hepatocellular carcinoma, YBX1 inhibits the maturation of miR-205 and miR-200b, leading to increased expression of ZEB1 and subsequently enhancing cell migration and invasion [42]. Furthermore, YBX1’s cytoplasmic activity promotes the tumorigenicity and metastatic potential of melanoma cells by facilitating EMT-like properties [111]. YBX1 interacts with G3BP1 and upregulates their downstream target SPP1, both in vitro and in vivo, resulting in activation of the NF-κB signaling pathway and the enhancement of cell adhesion, migration, and invasion in renal cancer [112]. Additionally, YBX1’s relevance to metastasis has been documented in various cancer types, including gastric cancer [113], skin squamous cell cancer [114], prostate cancer [115], ovarian cancer [116], and spinal chordoma [25].

**Figure 3:**
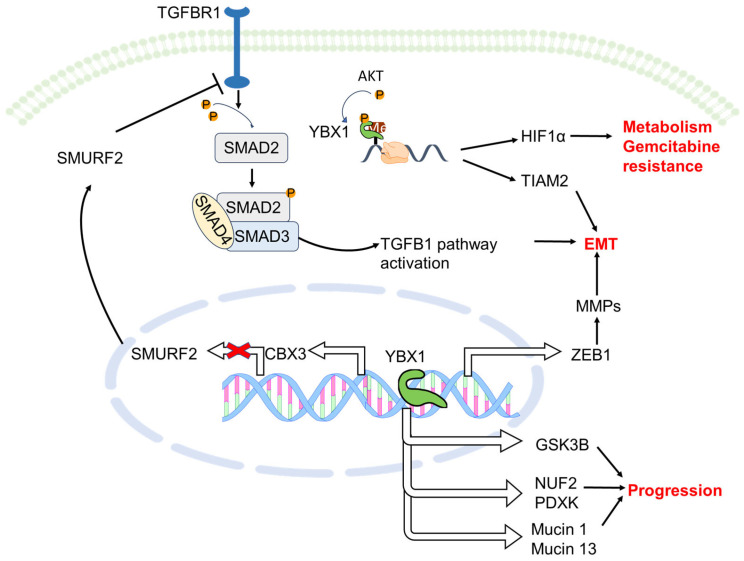
Role of YBX1 in pancreatic cancer. YBX1 was reported to promote EMT, proliferation, and progression of pancreatic cancer cells through its capability to regulate transcription and translation. Gasdermin-E interacted with and promoted YBX1 accumulation in nucleus, where YBX1 elevated mucin 1 and mucin 13 expression. Ultimately, pancreatic ductal adenocarcinoma cells escaped from digestive enzyme in pancreatic microenvironment [128]. The lncRNA antisense RNA1 of HIF1α upregulated HIF1α expression through promoting phosphorylation of YBX1 by AKT and thus enhanced glycolysis and gemcitabine resistance of pancreatic cancer cells [129]. YBX1 also promoted pancreatic ductal adenocarcinoma cell proliferation by transcriptionally increasing GSK3B expression which in turn regulated cyclin D1 and E1 levels [21]. circFOXK2 formed a complex with YBX1 and hnRNPK, elevating expression of onco-genic proteins, cell division cycle associated 1, and pyridoxal kinase, which contributed to pancreatic ductal adenocarcinoma progression [130]. LncRNA-BX111887 activated transcription of ZEB1 via recruiting YBX1 to its promoter region, which regulated E-cadherin and MMP2 expression and promoted EMT in pancreatic cells [131]. Moreover, knockdown of YBX1 remarkedly inhibited cell invasion with downregulation of MMPs in vitro [110]. Silencing of YBX1 led to a reduction in T cell lymphoma invasion and metastasis mRNA stability, which mediated EMT migration and invasion in pancreatic cancer cells [132]. Abbreviations: SMURF2: SMAD-specific E3 ubiquitin protein ligase 2; CBX3: chromobox protein homolog 3; TIAM2: T cell lymphoma invasion and metastasis; MMPs: Matrix metalloproteinases; NUF2: cell division cycle-associated 1; PDXK: pyridoxal kinase.

Recently, the spread of cancer to the bones is regarded as the visible manifestation of the specific molecular subtypes within PDAC, linked to poor prognoses [117]. Only several papers so far have reported the impact of YBX1 on pancreatic cancer progression and metastasis (Figure 3), despite its ubiquitous investigation in other cancers (Figure 2). While the role of YBX1 in bone metastasis has been extensively studied in various types of carcinomas, there is a notable lack of specific reports detailing its direct involvement in the metastasis of pancreatic cancer to the bone. Existing research has primarily focused on YBX1’s impact on bone metastasis in other types of cancer, elucidating its role and mechanisms in promoting or suppressing metastatic processes in those contexts.

For instance, in breast cancer with bone metastases, serum levels of secreted YBX1 (sYBX1) were found to correlate with extra-bone metastases and faster bone disease progression. However, it did not significantly impact overall survival or skeletal-related events in these patients [118].

In colorectal carcinoma (CRC), a specific circRNA, circNEIL3, has been identified as associated with metastasis repression by interacting with YBX1. This interaction leads to YBX1 degradation, suggesting circNEIL3 as a potential biomarker and therapeutic target in CRC due to its negative correlation with YBX1 levels and metastatic tendencies in CRC patients [119].

Moreover, acting as a transcription regulator, YBX1 mediates cell proliferation, invasion, and metastasis by upregulating the transcription of specific genes in different cancer contexts. These include FZD7 (Frizzled Class Receptor 7) in intrahepatic cholangiocarcinoma [120], Tumor-Associated Epithelial Mucin in lung adenocarcinoma [121], and FOXK1 (Forkhead Box K1) and CTP Synthase 1 in triple-negative breast cancer [122,123] (Figure 2).

At the level of translational regulation, YBX1 plays a pivotal role by binding to and enhancing the translation of several downstream genes, thereby promoting cell invasion and metastasis. For instance, in clear cell renal cell carcinoma, it stimulates the translation of Snail Family Transcriptional Repressor 1 [124], and in sarcoma cells, it enhances the translation of Hypoxia Inducible Factor 1 Subunit Alpha (HIF1α) [125]. Furthermore, YBX1 has been demonstrated to regulate the translation of both c-MYC and TGFβ1 mRNAs, which are associated with cell proliferation pathways [126,127].

YBX1’s interaction with multiple proteins and lncRNAs facilitates cancer cell migration, invasion, and metastasis in various cancers [73,105,133,134]. The lncRNA RP11-296E3.2 drives colorectal cancer by binding YBX1, activating STAT3 signaling, and fostering tumorigenesis and metastasis [135]. Targeting this RP11-296E3.2/YBX1 pathway could offer a promising approach for CRC diagnosis and treatment.

Moreover, studies have investigated the role of YBX1 in regulating metastasis-related processes in various cancers. For instance, in primary bone cancer, tRNA-derived small RNA fragments (tRFs) act as tumor suppressors, impeding pro-metastatic interactions. Notably, tRF-GlyTCC and RUNX2, regulated by miR-140 and associated with the YBX1 recognition site, play roles in metastatic processes [136]. Additionally, the investigation of lncRNA AC073352.1 in breast cancer revealed its upregulation in cancer tissues and its association with advanced stages. It interacts with YBX1, stabilizing its expression and influencing metastasis, suggesting AC073352.1 as a potential prognostic biomarker and therapeutic target in breast cancer [137].

In HCC, circASH2, a nuclear circRNA, functions as a suppressor of metastasis by regulating TPM4 and influencing the cytoskeleton structure. This regulation involves mRNA/precursor mRNA splicing and degradation, facilitated by the liquid–liquid phase separation of nuclear YBX1, providing insight into a precise regulatory mechanism in HCC progression [117].

### 3.5. Role of YBX1 in Deregulating Cellular Energetics

One notable characteristic of cancer cells is their altered carbohydrate metabolism [4]. Instead of relying on oxidative phosphorylation even in the presence of oxygen, cancer cells undergo a metabolic reprogramming known as aerobic glycolysis or the Warburg effect [138]. This metabolic shift provides tumor cells with various advantages for growth, metastasis, resistance to therapeutics, and survival in the harsh tumor microenvironment [139].

Studies investigating the role of YBX1 in cancer metabolism have demonstrated its involvement in regulating glycolysis. In bladder cancer, knockdown of YBX1 inhibits glycolysis, as evidenced by a decrease in glucose uptake and lactate secretion, which can be rescued by overexpression of HIF1α or Myc [140]. In another study, YBX1 is recruited to promote HIF1α translation by HIF1A-AS1 and lncRNA, thus mediating key glycolytic proteins, including Glucose transporter 1, hexokinase 2, pyruvate kinase muscle isozyme M2, and lactate dehydrogenase A, in pancreatic cancer [129]. Additionally, in breast cancer, knocking down YBX1 suppresses the protein expression of glycolysis signatures Enolase 1, Glucose-6-Phosphate Isomerase, and lactate dehydrogenase A, along with a reduction in lactate secretion [141].

THOC3, regulated by CCT proteins, partners with YBX1 to support PFKFB4 expression, promoting lung squamous cell carcinoma (LUSC) progression [142]. YBX1’s role in stabilizing PFKFB4 mRNA emphasizes its significance in this mechanism, highlighting its association with THOC3 (THO Complex Subunit 3) in LUSC development.

These findings underscore the role of YBX1 in modulating glycolytic pathways in various types of cancer, highlighting its potential as a target for therapeutic interventions aimed at disrupting cancer cell metabolism.

### 3.6. Role of YBX1 in Genome Instability and Mutation

The intricate machinery responsible for preserving DNA integrity possesses a remarkable capacity to detect and repair DNA damage, effectively preventing the occurrence of spontaneous mutations in each cell cycle. However, in the context of tumorigenesis, cancer cells often display an elevated mutation rate. They accumulate a collection of mutated genes that are essential for the initiation and progression of tumors [3,4,143,144]. Despite the genomic instability and mutation-related role of YBX1, its role in pancreatic cancer seems to be insufficient.

Accumulating evidence highlights the role of YBX1 in orchestrating genome instability. The level of YBX1 is inversely correlated with PTEN (Phosphatase And Tensin Homolog expression) [145], which is a critical factor involved in double-strand break repair and nucleotide excision repair [146]. Furthermore, YBX1 exerts a negative regulatory effect on p53 expression [147,148,149]. As a central tumor suppressor, p53 stabilizes the genome by coordinating various DNA damage-response mechanisms, including initiating cell cycle arrest to provide time for repair machinery to restore genome stability [150] (Figure 1).

Some other studies suggest a significant role of YBX1 in facilitating DNA damage repair and maintaining genome stability, ultimately preventing cancer cells from succumbing to death signals. MSH5 (MutS Homolog 5) plays a key role in the mismatch repair process, and the MSH4-MSH5 heterodimers are essential for homologous recombination repair of double-strand DNA breaks [151]. YBX1 has been identified as a binding partner for the promoter region of MSH5, effectively promoting the expression of MSH5 mRNA [152]. MajSAT RNA, under stressful conditions, hinders the movement of YBX1 into the cell nucleus, thereby diminishing its ability to repair DNA damage [153]. Interestingly, knockdown of YBX1 leads to a substantial increase in the phosphorylation of H2AX, indicating the occurrence of DNA damage in cells [74]. Further investigation is needed to gain a better understanding of the role of YBX1 in the regulation of DNA damage repair.

Recently, in GBM, DARS1-AS1 influences malignant traits in GBM/GSCs, notably interacting with YBX1 to regulate key genes involved in tumor progression and DNA repair [29]. Targeting the DARS1-AS1/YBX1 axis holds promise as a therapeutic approach to sensitize GBM to radiation and HR deficiency-targeted therapies. Fibrillarin (FBL), known for RNA methylation, unveils a role in DNA damage response where it interacts with YBX1, facilitating nuclear accumulation upon DNA damage [154]. FBL’s influence on YBX1 enhances BRCA1 expression, revealing potential therapeutic targets to combat chemoresistance in cancer by understanding their interaction in DNA damage responses. In glioma stem cells (GSCs), inhibiting PLK1 induces apoptosis and DNA damage by phosphorylating YBX1 at serine 174 and serine 176, impairing its nuclear translocation [74]. This highlights YBX1’s crucial role as PLK1 inhibition triggers cell death and DNA damage via YBX1 phosphorylation, offering insight into potential glioma treatment mechanisms.

One extensively studied epigenetic modification is the methylation of cytosine at the fifth position (5mC), which frequently occurs at CpG islands proximal to gene promoters and is involved in the regulation of gene expression [155]. In addition to DNA, RNA is also a target of epigenetic modification. An important RNA modification is 5mC, which is known to regulate mRNA stability and translocation. YBX1, with its highly preserved Cold Shock Domain, has long been identified as a DNA/RNA binding protein. Increasing evidence illustrates the ability of YBX1 to bind to various types of RNA, as summarized in Table 1. One of the mechanisms driving YBX1 to bind RNA is epigenetic-dependent binding, in which YBX1 serves as a reader of 5mC, along with ALYREF (Aly/REF Export Factor) [156,157].

RNA-BisSeq data revealed that 5mC hypermethylation is a frequent event in urothelial carcinoma of the bladder, and hypermethylated mRNAs are enriched in oncogenic pathways (JAK–STAT, ERBB, PI3K–AKT, VEGF, EMT, and ERK–MAPK) [156]. The amino acid residue W65 within the Cold Shock Domain of YBX1 is responsible for recognizing 5mC. YBX1 acts as a 5mC reader and recruits ELAV-Like RNA Binding Protein 1 to stabilize HBGF (Heparin Binding Growth Factor) mRNA, thus facilitating metastatic tumor progression [156]. In prostate cancer, analyzed via electrophoretic mobility shift assay and pulldown assay, YBX1 can bind to 5mC-modified androgen receptor (AR) mRNA but not the unmodified one. Moreover, silencing of YBX1 leads to a decrease in AR mRNA, which is not related to transcriptional activity. These results, together with the established role of YBX1 in mRNA stability, suggest that YBX1 binds to the m5C of AR mRNA and enhances its stability, further promoting prostate cancer proliferation and invasion [158]. In gastric cancer, YBX1 serves as a 5mC reader and stabilizer of *ORAI2* mRNA, resulting in the upregulation of E2F1, promoting peritoneal metastasis and colonization of gastric cancer [159]. In breast cancer, YBX1 not only initiates KLF5 (Kruppel-Like Factor 5) transcription but also recognizes 5mC-modified *KLF5* mRNA to stabilize it, ultimately promoting tumor progression [160]. Additionally, YBX1 reads m5C PEBP1 (Phosphatidylethanolamine Binding Protein 1) mRNA in clear cell renal cell carcinoma [161] and 5mC keratin 13 mRNA in gynecologic cancers [162], preventing their decay, which contributes to tumor progression.

Other RNA modifications, such as 6mA [163,164,165,166,167,168] and 1mA [169,170], have also been extensively investigated in various post-transcriptional processes. Although YBX1 does not directly bind to m6A, it is recruited to m6A sites through binding to insulin-like growth factor 2 messenger RNA binding proteins and plays a crucial role in *MYC* and *BCL2* mRNA stabilization [62].

Furthermore, recent studies have linked the overexpression of m6A/m5C/m1A regulatory genes, including YBX1, to poor prognosis and distinct immune microenvironments in HCC and prostate cancer [171,172,173]. Further research is needed to deepen our understanding of the complex relationship between epigenetic modifications and immune evasion, which collectively contribute to the exacerbation of tumors.

**Table 1:** RNA binding partners of YBX1.

Effect	Partner	Name	Reference	Cancer Type
**Bind to and stabilize YBX1 mRNA or enhance S102 phosphorylation leading to facilitating translation or transcription of target genes**	Long noncoding RNA (lncRNA)	HOXC-AS3	[174,175]	Lung cancer, pancreatic cancer, esophageal squamous cell carcinoma, breast cancer, acute myeloid leukemia, clear cell renal cell carcinoma, hepatocellular carcinoma (HCC), skin cancer
		HIF1A-AS1	[129]	
		MIR31HG	[94]	
		LINC00857	[176]	
		linc02042	[177]	
		AC073352.1	[137]	
		GAS6-AS1	[178]	
		MILIP	[124]	
		USP2-AS1	[179]	
		HUMT	[122]	
		BASP1-AS1	[180]	
		DSCAM-AS1	[181]	
		LINC00941	[182]	
		HOTAIR	[183]	
		GAS5	[184]	
		SNHG6	[185]	
		linc00665	[186]	
		PIK3CD-AS2	[187]	
	Circular RNA (circRNA)	circACTN4	[120]	Intrahepatic cholangiocarcinoma, nasopharyngeal carcinoma, HCC
		circIPO7	[106]	
		hsa_circ_0062682	[188]	
**Bind to and inhibit YBX1 or nuclear translocation**	lncRNA	Linc01612	[189]	HCC, lung adenocarcinoma, colorectal cancer
		LINC00472	[190]	
		TP53TG1	[191]	
		HITT	[192]	
	circRNA	circNEIL3	[119]	Colorectal carcinoma, gastric cancer
		circFAT1(e2)	[193]	
	Micro RNA (miRNA)	miR-6509-5p	[194]	Gastric cancer, diffuse large B-cell lymphoma, malignant pleural mesothelioma, cervical carcinoma, breast cancer, renal carcinoma
		MiR-216a	[195]	
		miR-137	[196,197]	
		miR-375	[198,199,200]	

## 4. Role of YBX1 in Tumor Microenvironment

### 4.1. YBX1 in Inducing Angiogenesis

Similar to normal tissues, tumors also require the ability to acquire nutrients, oxygen, and eliminate metabolic waste products. This process is facilitated by the formation of new blood vessels induced through angiogenesis. During tumor progression, an “angiogenic switch” is typically activated, leading to the continuous development of new blood vessels that support the growth of tumor tissue [201]. YBX1 has been reported to bind to the promoters of angiogenesis-related genes such as *ANGPT4* (*Angiopoietin 4*), *ANGPTL3* (*Angiopoietin Like 3*), and *VEGFA* (*Vascular Endothelial Growth Factor A*), thereby activating their expression at both the mRNA and protein levels. This activation, in turn, promotes the formation of capillary tubes by human umbilical vein endothelial cells (HUVECs) in vitro [186]. HIF1α is a well-known inducer of angiogenesis, and it has been observed that YBX1 binds to HIF1α mRNA, thereby promoting its translation [125].

As a potential RNA binding protein, YBX1 enhances the angiogenic ability of HUVECs by binding and promoting AC073352.1, a lncRNA significantly upregulated in breast cancer, sorting it into exosomes and mediating angiogenesis [137]. YBX1 also regulates angiogenesis in fibroblasts by sorting miR-133 into exosomes [202].

Furthermore, YBX1 plays a role in regulating angiogenesis through its interactions with other molecules. In ovarian carcinoma-derived microvascular endothelial cells, YBX1 competes with miR-376A for binding to *MEG3* (*maternally expressed gene 3*) lncRNA. This competition affects the expression of *RASA1* (*RAS p21 protein activator 1*), a key suppressor of tube formation whose expression is targeted and inhibited by miR-376A. Elevated levels of YBX1 lead to the release of miR-376 from MEG3, which in turn reduces RASA1 expression, ultimately impacting angiogenesis [203].

Recent research has also highlighted the role of YBX1 in the secretome. When comparing YBX1-overexpressed Madin-Darby Canine Kidney (MDCK) cells to regular MDCK cells, the secretome of YBX1-overexpressing MDCK cells was found to contain 47 enriched proteins, including many that are known to be involved in angiogenesis. These proteins include proteases like ADAM9 (ADAM Metallopeptidase Domain 9) and ADAM17, inhibitors such as SERPINC1 (Serpin Peptidase Inhibitor Clade C Member 1), as well as stimulatory and growth factors like CSF1 (Colony-Stimulating Factor 1), VGF (VGF Nerve Growth Factor Inducible), and NGF (Nerve Growth Factor) [204]. Intriguingly, elevated levels of YBX1 in exosomes originating from gastric cancer are internalized by HUVECs, leading to increased expression of angiogenic factors, including IL-8, VEGF, ANGPT1, and MMP-9, at both the mRNA and protein levels [205]. However, there does not appear to be any research findings related to YBX1 and angiogenesis in pancreatic cancer as of yet.

In osteosarcoma, VEGF165 promotes tumor progression, while VEGF165b opposes it; YBX1, a critical splicing factor, upregulates VEGF165 but downregulates VEGF165b, impacting both their levels and the disease course [206]. Targeting YBX1 may modulate VEGF165 and VEGF165b concurrently, offering a potential avenue for therapeutic intervention in osteosarcoma.

### 4.2. YBX1 in Avoiding Immune Destruction

Tumor cells predominantly express Th2 type cytokines, such as IL-4, IL-6, and IL-10 [207,208] which aid tumor cells in evading immune destruction by inhibiting the differentiation of CD8+ T cells [209]. The expression level of YBX1 shows a significant positive correlation with CD4+ Th2 cells across twenty-six different cancer types. It also promotes IL-4 expression in oral squamous cell carcinoma, suggesting YBX1’s involvement in shielding cancer cells from immune responses [210]. Furthermore, a high level of YBX1 is linked to the infiltration of macrophage M2 type and the expression of T cell exhaustion markers like IDO1 and CTLA4. This association accelerates the progression of luminal breast cancer [211].

Another crucial immune checkpoint is the PD1/PDL-1 axis, which normally functions to control excessive immune cell activation and prevent autoimmune diseases. However, in the tumor microenvironment, cancer cells exploit this axis to escape immune surveillance [212]. Overexpressed PD-L1 on cancer cells binds to PD-1 on tumor-infiltrating lymphocytes (TILs), leading to the inhibition of the TCR signaling cascade [213,214], consequently hindering T cell activation. Importantly, it is not just cancer cells; other elements within the Tumor microenvironment, including macrophages, dendritic cells (DCs), activated T cells, and cancer-associated fibroblasts, also express PD-L1 [215]. These components collaborate to create an immunosuppressive microenvironment that supports tumor growth. It has been difficult to find reports on the role of YBX1 in PD-L1 expression in pancreatic cancer.

Recent studies have furnished evidence highlighting the role of YBX1 in immune evasion. YBX1 can bind to the PD-L1 promoter and transcriptionally boost PD-L1 expression, which significantly contributes to T cell inactivation and evasion of immune surveillance [216,217], as depicted in Figure 2. Combining YBX1-targeted drugs with immune checkpoint blockade may present a promising approach to enhance the effectiveness of cancer treatment.

The TGFβ pathway plays a crucial role in facilitating the evasion of immune responses through multiple mechanisms [218]. In lung cancer, it has been documented that YBX1 mediates the upregulation of TGFBR1, consequently enhancing TGFβ1 signaling [104]. Furthermore, YBX1 promotes the translation of TGFβ1 mRNA in proximal tubule cells [219], and knocking down YBX1 reduces the downstream signaling molecules of TGFβ1 in renal cell carcinoma [220]. Despite the established association between YBX1 and TGFΒ1 signaling, the exact involvement of the YBX1/TGFΒ1 axis in the regulation of immune responses remains a compelling area for further investigation.

In a different context, it has been observed that activated T cells from patients with SLE have a compromised ability to enhance or sustain YBX1 expression upon activation. As a result, these activated T cells undergo apoptosis, potentially leading to prematurely terminated immune responses against pathogens. The accumulation of cellular debris in this scenario might contribute to initiating and perpetuating the autoimmune process [79]. These findings underscore the complex regulatory role of YBX1 in immune responses.

Lately, several molecules that influence cancer immunity by interacting with YBX1 have been identified. For instance, in liver hepatocellular carcinoma, a novel Anoikis-related score was developed, accurately predicting immunological activity, gene alterations, and medication sensitivity. SPP1 emerged as a significant predictor for immunotherapy outcomes, influencing LIHC cell behavior, with a potential YBX1/SPP1 axis identified, offering insights for improved patient care and treatment strategies [221]. Immunity-related GTPase M (IRGM), a critical player in immune regulation, interacts with YBX1 to facilitate PD-L1 transcription, suppressing CD8+ T cell infiltration in HCC and contributing to cancer progression [222]. This interaction unveils a potential therapeutic avenue by combining IRGM inhibition with immune checkpoint inhibitors, highlighting YBX1’s pivotal role in the regulation of immune responses in hepatocellular carcinoma. Lnc-SOX9-4, an upregulated RNA in CRC, is linked to poor prognosis and influences immune cell infiltration. Its interaction with YBX1 significantly impacts CRC progression, stabilizing YBX1 and promoting aggressive cancer behavior [223]. KIF14’s heightened presence in CCA correlates with aggressive traits, impacting survival and chemotherapy response. Its interaction with the G3BP1/YBX1 complex activates NF-κB, fostering metastasis, chemo-resistance, and an immunosuppressive environment, highlighting YBX1’s pivotal role in CCA progression and prognosis [134].

### 4.3. YBX1 in Tumor-Promoting Inflammation

Tumor microenvironments are marked by the dense infiltration of cells from both the innate and adaptive immune systems, creating an inflammatory-like condition akin to that seen in normal tissues. Initially, these immune responses were thought to signify the immune system’s effort to eliminate tumors. However, mounting evidence now indicates that inflammation can also function as a factor that promotes tumor growth. It provides the tumor microenvironment with bioactive molecules that stimulate tumor cell proliferation, angiogenesis, invasion, and metastasis. Additionally, inflammation has been implicated in driving the genetic evolution of tumors towards a more malignant state [4].

Inflammation induced by smoking seems to lead to epigenetic changes that may contribute to smoking-related pancreatic cancer [224]. Exposure to cigarette smoke extract was observed to cause an increase in the overexpression of YBX1, which subsequently led to the upregulation of CBX3 (Chromobox 3) in pancreatic cancer cells. Chromobox 3 appeared to play a role in enhancing the progression of pancreatic cancer, possibly by suppressing the expression of SMAD-specific E3 ubiquitin protein ligase 2 and promoting the activation of the TGFβ signaling pathway [225].

A growing body of evidence links YBX1 to the regulation of inflammation and the facilitation of tumor progression. IL-6 is a proinflammatory cytokine that has been implicated in promoting breast cancer metastasis by activating the notable JAK/STAT3 signaling pathway [226,227]. Inflammatory stimuli induce expression of YBX1. This heightened expression of YBX1 is associated with drug resistance to JAK inhibitors in cases of myeloproliferative neoplasia [228]. YBX1 has been shown to bind to and stabilize IL-6 mRNA through RNA Immunoprecipitation (RIP) assay, resulting in increased IL-6 expression. Notably, it has been observed that IL-6 stimulation can induce YBX1 expression, suggesting the existence of a positive feed-forward loop that contributes to the process of EMT in breast cancer cell lines [229].

CCL5 (C-C Motif Chemokine Ligand 5) is a proinflammatory chemokine primarily expressed by T cells and monocytes, playing a crucial role in recruiting leukocytes to sites of inflammation. It acts as a chemoattractant for various cell types, including T cells, eosinophils, basophils, monocytes, natural killer (NK) cells, dendritic cells, and mastocytes. YBX1 has been identified as a target gene of CCL5 in a positive regulatory relationship observed in smooth muscle cells and immune cells such as T cells and monocytes. In the tumor microenvironment, CCL5 promotes cancer progression [230,231,232] implying a connection between YBX1 and tumor-promoting inflammation.

Furthermore, YBX1-deficient macrophages exhibit impaired cell polarization and function, including reduced proliferation and nitric oxide production, decreased phagocytic activity, and an inability to upregulate IL-10 and CCL5 expression in response to inflammatory stimuli. This ultimately leads to a failure in resolving inflammation [233]. Such dysregulation may contribute to the maintenance of a chronic inflammatory microenvironment at the tumor site, promoting cancer progression.

### 4.4. Role of YBX1 in Neural Input

Recently, the importance of neural input in cancer, especially in the cancer microenvironment, has been increasingly emphasized [234,235]. In particular, in the tumor microenvironment, neural inputs are beginning to be recognized as a new factor that should be integrated with the immune environment for cancer treatment [236,237].

YBX1 is highly expressed not only in other tissues but also in neural tissues. For example, YBX1 mRNA exhibits high levels in the brain, as well as in other tissues such as the heart, muscle, liver, lung, and adrenal gland [238]. In contrast, it was present in lower amounts in the thymus, kidney, bone marrow, and spleen. Examination of YBX1 immunolabeling, along with dual-labeling using the neuronal marker NeuN in rat brain tissue, demonstrated that YBX1 is primarily expressed in neurons. Specifically, this expression was prominent in the dentate gyrus, the pyramidal cell layer of the cornu ammonis, layer III of the piriform cortex, and across all layers of the parahippocampal cortex [239]. In addition, within the hilus of the hippocampus, isolated neurons exhibited YBX1 expression.

In the cytoplasm of dorsal horn neurons, a circRNA, CircAnks1a facilitates YBX1 nuclear translocation through promoting the interaction between YBX1 and transportin-1. In the nucleus, circAnks1a binds directly to the Vascular Endothelial Growth Factor B (Vegfb) promoter and increases recruitment of YBX1 to the Vegfb promoter, thereby elevating transcription of Vegfb. Vegfb upregulation ultimately increases the pain-like hypersensitivity response of dorsal horn neurons [240]. PRC2 interacts with YBX1, and in mouse embryos in vivo, YBX1 plays a crucial role in forebrain specification and limiting mid-hindbrain growth [241]. Within neural progenitor cells, YBX1 is required in both self-renewal and neuronal differentiation. Mechanistically, YBX1 extensively overlaps with PRC2 binding across the genome, influencing PRC2 distribution and reducing H3K27me3 levels. The downregulated genes in YBX1 knockout neural progenitor cells that bind to YBX1 were enriched in functions related to nervous system development, neuron differentiation, synapse transmission, axon guidance, glutamate signaling, and BDNF signaling, indicating that Ybx1 promotes the expression of genes related to brain development, neurogenesis, and neuronal function. Furthermore, YBX1 from neural stem cell-derived extracellular vesicles inhibited neuronal pyroptosis and ischemia/reperfusion injury of rats by interacting with IGF2BP1 and then promoting the stability of m6A-modified GPR30 mRNA, which in turn inhibited the activation of the NLRP3 inflammasome [242].

There are many reports that YBX1 is associated with neurodegenerative disease. Overexpression of YBX1 has the potential to activate the AKT/GSK3B pathway in PC12 cells subjected to oxygen–glucose deprivation/reoxygenation (OGD/R) [243]. Consequently, YBX1 may provide protection against OGD/R-induced damage in PC12 cells by activating the AKT/GSK3B signaling pathway. YBX1, which might also have an impact on stress granule formation, is regarded as a pathological marker associated with amyotrophic lateral sclerosis [244]. TARDBP (TAR DNA Binding Protein) and FUS (FUS RNA Binding Protein) are mRNA binding proteins linked to neurodegenerative diseases. The age-related cyclic formation and dissolution of stress granules in neurons, which are regulated by YBX1 and G3BP1, may contribute to protein aggregation, a hallmark of neurodegenerative diseases [245].

A large amount of research has been conducted on the role of YBX1 in cancers related to the brain and nervous system. The analysis of protein–protein interactions, utilizing integrated proteomics and transcriptomics data for transcription factors and invasion-associated proteins in cell invasion processes (drawn from a separate GBM dataset), identified YBX1 [246]. Inhibiting YBX1 led to decreased GBM tumor cell invasion and growth, both in monolayer cultures and soft agar [247]. Additionally, it delayed the onset of tumors in mice. Importantly, YBX1 inhibition improved sensitivity to temozolomide in both adult and pediatric GBM models, and this effect was not dependent on O6-methylguanine-DNA methyltransferase.

YBX1 emerged as a pivotal interactor with key proteins associated with cell invasion in GBM. miR-137 is associated with GB formation and inhibits the expression of YBX1 and other molecules including c-Kit, AKT2, CDC42, CDK6, and TGFβ2 [248]. YBX1 protein displayed significant overexpression in GB and played a crucial role in activating both mTORC1 and mTORC2 signaling pathways [249]. Mechanistically, YBX1 interacted with the 5′UTR of CCT4 (Chaperonin-Containing TCP1 Subunit 4) mRNA, facilitating the translation of CCT4, a key component of the CCT chaperone complex. This, in turn, activated the mTOR signaling pathway by promoting the folding of MLST8 (MTOR-Associated Protein, LST8 Homolog). Additionally, YBX1 engaged in autoregulation by binding to its own 5′UTR, resulting in sustained activation of the mTOR signaling pathway.

The role of YBX1 in glioma has also been reported. Kindlin-2 and GOLPH3 (Golgi phosphoprotein 3) proteins are associated with glioma, and it appears that YBX1 protein is involved in the increased expression of EGFR and the activation of mTOR in this context. For example, elevated Kindlin-2 expression correlated with higher pathological grades and poorer prognosis [250]. In both in vitro and in vivo settings, Kindlin-2 promoted the motility and proliferation of glioma cells. Kindlin-2 also formed a transcriptional complex with YBX1 and β-catenin, which was bound to the EGFR promoter and augmented transcription. When GOLPH3 was down-regulated, it resulted in a substantial reduction in the levels of YBX1 and mTOR activity [251], which are both crucial for glioma cell migration and invasion. Conversely, the levels of YBX1 and mTOR activity increased when GOLPH3 was overexpressed.

YBX1 levels are heightened in medulloblastoma and play a role in promoting proliferation in Sonic Hedgehog-dependent cerebellar granule neuron progenitor cells and medulloblastoma cells [252]. Depleting YBX1 in both Sonic Hedgehog and Group 3 medulloblastoma not only decreases proliferation but also synergizes with radiation treatment, primarily due to distinct response dynamics [253]. The silencing of YBX1 using shRNA followed by ionizing radiation primarily induces a repair mechanism reliant on Non-Homologous End Joining. This results in faster resolution of γH2AX, premature cell cycle re-entry, bypassing of checkpoints, reduced proliferation, and an increased tendency towards senescence.

**Figure 4:**
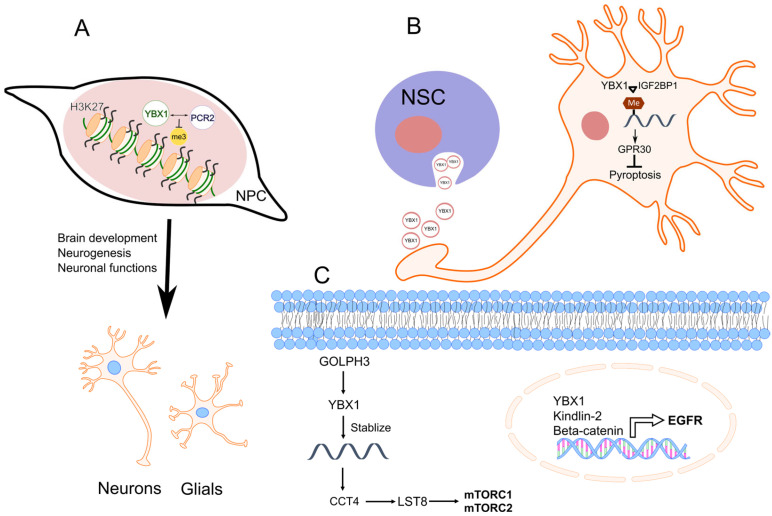
YBX1 as a neural input. (**A**) YBX1 promotes neurogenesis and differentiation of neural progenitor cells (NPC). (**B**) Extracellular vesicle from neural stem cells (NSC) carrying YBX1 suppresses neuronal pyroptosis in rats. (**C**) YBX1 activates mTOR and EGF signaling in glioma.

The above-mentioned evidence has shed light on the role of YBX1 as a neural input, as depicted in Figure 4. Neo-neurogenesis is gradually gaining attention for its role in the tumor microenvironment and cancer progression. Although neural plasticity has been reported to be important in pancreatic cancer [254], there are still no reports on the role of YBX1 in this process. It appears that future research should actively explore the role of YBX1 in neurogenesis and its relevance to various neural receptor molecules associated with cancer.

### 4.5. Effects of YBX1 on Extracellular Matrix

YBX1 assumes a pivotal role within the extracellular matrix (ECM) of tumors, orchestrating a complex interplay that shapes the ECM’s composition, remodeling dynamics, and interaction with cancer cells. Through direct modulation of protein expression, notably collagens and fibronectin [255,256], YBX1 actively contributes to the formation of a dense and rigid ECM scaffold, fostering an environment conducive to malignant cellular behaviors.

Its regulatory influence extends to the intricate dance between matrix metalloproteinases (MMPs) and TIMPs (tissue inhibitors of metalloproteinases), where YBX1 delicately adjusts ECM breakdown and restructuring [110], akin to a meticulous architectural designer refining structural elements.

Beyond ECM modulation, YBX1 exerts a profound impact on integrin signaling pathways within cancer cells [257]. This regulatory role resembles a conductor guiding the coordinated actions of musicians, influencing critical cellular processes including survival, proliferation, and adhesion. YBX1’s influence on these pathways dictates the cellular response to the ECM, influencing the invasive behavior of cancer cells towards metastasis.

Strategies targeting YBX1’s interactions with ECM components present promising avenues for therapeutic intervention. Disrupting YBX1’s influence on ECM construction could impair the supportive scaffold, hindering tumor cell behavior. Similarly, inhibition of YBX1-mediated modulation of ECM remodeling enzymes might alter the architectural dynamics of the ECM, impeding migration. Moreover, interventions aimed at disrupting YBX1-mediated integrin signaling could sever the essential link between cancer cells and the ECM, potentially halting malignancy progression.

## 5. Targeting YBX1 in Cancer Therapy Research and Development

YBX1 expression levels and subcellular localization could serve as valuable prognostic markers, helping predict tumor aggressiveness and recurrence risk. For instance, high nuclear YBX1 might indicate poor prognosis, while cytoplasmic localization could suggest a less aggressive tumor [258]. A comprehensive meta-analysis has unveiled a substantial connection between increased YBX1 expression and unfavorable tumor characteristics, such as poor differentiation, larger tumor size, and the presence of lymph node metastasis [259]. Additionally, high levels of YBX1 in the cell nucleus have been strongly linked to decreased overall survival rates. Identifying patients with tumors harboring specific YBX1 mutations or interacting proteins could pave the way for personalized treatment options. This could involve tailoring therapy based on the individual cancer’s dependence on YBX1’s oncogenic or tumor-suppressive functions [260].

Ongoing clinical trials are currently assessing the prognostic value of YBX1, with a specific focus on myeloproliferative neoplasms, in order to validate its clinical relevance (Table 2). Furthermore, YBX1 has been identified as one of the immunogenic proteins targeted in the STEMVAC trial for breast cancer and lung cancer, as outlined in Table 2. STEMVAC is a DNA plasmid-based vaccine (STEMVAC) encoding Th1 selective epitopes from five antigens associated with breast cancer stem cells (MDM2, YB1, SOX2, CDC25B, CD105) which may be beneficial in helping the body to build an effective immune response to kill tumor cells. STEMVAC finished phase 1 with the result of high-level persistent type I T cell responses at the 300mcg dose in early-stage triple-negative breast cancer [261] and now is recruiting participants for phase 2 trials and expanding to patients with advanced-stage (III-IV) triple-negative breast cancer (Table 2). These trials underscore the growing interest in exploring YBX1 as a therapeutic target.

Understanding YBX1’s role in resistance to existing therapies could guide the development of more effective drug combinations. For instance, if a tumor shows upregulation of YBX1-mediated drug efflux pumps, co-administering an YBX1 inhibitor might overcome resistance [262,263]. While its well-established oncogenic role in diverse cancers remains prevalent, recent revelations of context-dependent tumor-suppressive functions add complexity to therapeutic pursuits. Researchers have suggested diverse strategies. For example, Nuclear translocation inhibitors aim to impede YBX1’s pro-tumorigenic activity in the nucleus by targeting YBX1-interacting proteins involved in nuclear transport [264]. Proteasome degradation emerges as a potential avenue, with small molecules inducing YBX1 ubiquitination and subsequent degradation being investigated [265,266]. Exploration of RNA interference therapies, such as siRNA and antisense oligonucleotides, seeks to silence YBX1 expression, especially in YBX1-dependent cancers [47]. Synthetic lethality approaches aim to identify genes whose loss is lethal only in the presence of YBX1 overexpression, offering potential targeted therapies [267]. Studying YBX1 interactions with other key cancer-related proteins could identify novel drug targets beyond YBX1 itself. This could lead to the development of more targeted and potentially less toxic therapies [268].

Azopodo-phyllotoxin SU056, a small molecule, directly binds to YBX1 and inhibits its expression, leading to cell cycle arrest and apoptosis, which has been tested in ovarian cancer cells [116]. Fisetin is also a small molecule that elicits the ability to bind and inhibit AKT-YBX1 interaction and, thus, inhibit EMT in prostate cancer [269]. Furthermore, Fisetin renders melanoma tumor growth both in vitro and in vivo through inhibiting RSK kinase activity and YBX1 total protein levels, as well as the S102 phosphorylated form [270]. CX-5461 is a small molecule inhibitor that targets RNA polymerase I, and it has shown potential to disrupt YBX1 activity [271]. TAS0612 elicits the inhibition effect on AKT, p70S6K, and p90RSK and thereby inhibits YBX1 phosphorylation and reverse antiestrogen resistance in breast cancer [32].

2,4-dihydroxy-5-pyrimidinyl imidothiocarbomate (DPI) has been shown to hinder YBX1 nuclear translocation and its downstream target genes, which in turn inhibits breast cancer cell proliferation, migration, invasion, and metastasis [272]. Similarly, Sesquiterpene lactone 6-O-angeloylplenolin suppresses YBX1 nuclear translocation, thus downregulating MDR1 expression in colon carcinoma [273].

7-hydroxyindirubin potentially inhibits YBX1 expression in the nucleus and thus sensitizes HepG2 cells to actinomycin D treatment [274]. BEZ235 is a PI3K/mTOR inhibitor that can inhibit YBX1 expression, suppress colorectal cancer cell proliferation, and promote radiation cytotoxicity both in vitro and in vivo [275]. In another study on HER2-positive breast cancer, aloe-emodin (AE) extracted from rhubarb roots showed the ability to suppress the ILK/AKT/mTOR axis and thus reduce YBX1 expression and elicit positive anticancer activity [276].

Despite promising strategies, challenges persist. YBX1’s context-dependent duality demands personalized therapeutic approaches, considering its varied roles across cancer types and cellular contexts. Developing YBX1-specific drugs while avoiding effects on other Y-box family proteins remains a challenge, preventing unintended side effects. Overcoming delivery barriers and potential drug resistance mechanisms requires further investigation. Future directions hold substantial promise:

Leveraging the tumor-suppressive functions of cytoplasmic YBX1 in specific cancers represents a novel therapeutic avenue. Innovative drug delivery systems, including nanoparticles, offer potential enhancements in targeted drug delivery overcoming tissue barriers [277]. Combining YBX1-targeting drugs with conventional therapies or inhibitors of YBX1-interacting proteins may present synergistic effects and combat resistance. The integration of computational modeling to predict YBX1–drug interactions and design more effective targeted therapies holds significant potential.

These molecular and compound-based strategies hold significant potential for suppressing YBX1 activity and mitigating its downstream effects, as summarized in Table 3.

Given the potential of YBX1 as a therapeutics target, certain limitations should be acknowledged. YBX1 mRNA exhibits high levels not only in the brain but also in other tissues such as the heart, muscle, liver, lung, and adrenal gland [238], suggesting a physiological role of YBX1. Its involvement in pregnancy is noteworthy, as both increases and decreases in YBX1 expression can contribute to diverse pregnancy-related complications [278]. Moreover, YBX1 plays a crucial role in the transcriptional and translational regulation across a variety of cellular activities. This implicates the double-edged sword significance of targeting YBX1 to be considered for successful YBX1-targeted therapy. Alternative approaches to target YBX1, with oligonucleotide-based methods such as siRNA and miRNA, along with virotherapy, show promise in cancer treatment. However, the effective delivery of siRNA to tumor cells within the human body poses a substantial challenge. Unmodified siRNA faces rapid degradation in the bloodstream, struggles to penetrate cells, and may trigger an immunogenic response. Meanwhile, virotherapy faces limited viral replication efficacy and host immune response that hinder its development. Addressing these obstacles is imperative in optimizing YBX1-targeted therapy for cancer treatment.

## 6. Perspective

**Figure 5:**
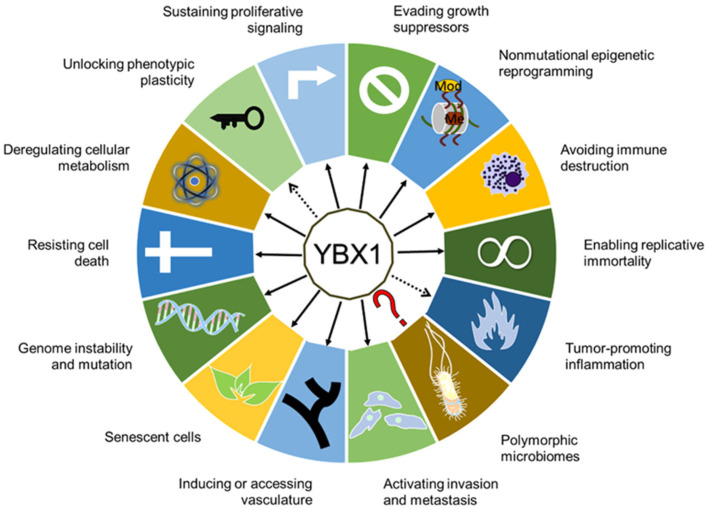
Involvement of YBX1 in hallmarks of cancer. YBX1 exhibits effects on almost all hallmarks of cancer which are proposed by Hanahan et al., [2,3,4].

In recent years, YBX1 has gained increasing attention in the field of cancer research due to its significant role in tumor initiation and progression. Extensive evidence has implicated YBX1 in various cancer hallmarks, but certain aspects of its involvement in cancer remain poorly understood, necessitating further investigation, as illustrated in Figure 5.

One particularly intriguing area of research pertains to the relationship between YBX1 and tumor-promoting inflammation. While studies have demonstrated that YBX1 can upregulate proinflammatory molecules like IL-6 and CCL5 in specific cell types, the overall impact of YBX1 on the inflammatory microenvironment associated with tumors remains unclear. It is crucial to decipher the precise mechanisms through which YBX1 influences the inflammatory milieu within tumors as this knowledge could offer valuable insights into cancer progression and potential therapeutic strategies.

Furthermore, emerging evidence suggests a potential link between YBX1 and phenotypic plasticity in cancer. This phenomenon enables cancer cells to evade differentiation and maintain a partially differentiated progenitor-like state. However, the exact contribution of YBX1 to this process and its implications for tumor heterogeneity and therapeutic responses necessitate further exploration. Understanding how YBX1 influences phenotypic plasticity could illuminate the mechanisms underpinning tumor progression and open up new avenues for targeted therapies.

A recent update by Hanahan in 2022 has introduced an emerging factor with a profound impact on cancer initiation and progression, termed the polymorphic microbiome [3]. The microbiome has been implicated in various aspects of cancer, including tumor development, immune modulation, and inflammation. Given YBX1’s role in immunity and inflammation, it is compelling to investigate the relationship between YBX1 and the tumor microbiome and, ultimately, how their interplay affects cancer progression.

While numerous studies support the oncogenic role of YBX1 in various cancers, promoting proliferation, migration, and chemoresistance [260,262], contrasting reports have emerged suggesting its tumor-suppressive functions. For example, there are reports suggesting that YBX1 behaves akin to rapamycin, hindering the translation of specific transcripts. This action leads to resistance against AKT1-induced oncogenic transformation in chicken embryo fibroblasts. [279,280]. Increased YBX1 expression in breast cancer suppresses cell proliferation by impeding the translation of growth-related mRNAs and concurrently enhancing mesenchymal gene expression, promoting cell survival in anchorage-independent conditions [40,41]. This apparent controversy likely stems from several factors such as cellular context, subcellular localization, and post-translational modifications.

Therefore, future research on YBX1 in cancer should consider these contextual factors to reconcile the seemingly contradictory findings. Investigating the interplay between specific cancer types, subcellular localization, and post-translational modifications will likely yield a more nuanced understanding of YBX1’s multifaceted role in tumorigenesis.

## 7. Conclusions

Despite the well-established role of YBX1 in cancer, certain aspects of its involvement in tumor-promoting inflammation, phenotypic plasticity, and interactions with the polymorphic microbiome remain uncertain. The potential of YBX1 in neurogenesis and its connection to neoneurogenesis also holds promise for future cancer research and application. Additionally, the increasing burden of PDAC, with a stagnant 5-year survival rate of 9.9% over the last three decades in China [69], underscores the urgent need for research and the development of more effective treatment options. Hence, further experimental studies are crucial to uncover the precise mechanisms and functional consequences of YBX1 in these contexts. Such investigations will not only deepen our understanding of cancer biology but also hold significant potential for the development of innovative therapeutic strategies targeting YBX1 in cancer. Moreover, considering YBX1’s diverse roles in various biological processes, it is essential for research in YBX1-targeted cancer treatment to proceed thoughtfully. This approach aims to maximize therapeutic effectiveness while minimizing potential side effects.

## Figures and Tables

**Table 2:** Current clinical trials anchoring YBX1.

Study Title	Clinical Trial ID	Drug in Trial	State	Aim	Disease
**Ruxo-BEAT**	2013-002132-25	Ruxolitinib	Ongoing	To prospectively validate Ybx1 as a diagnostic and putative prognostic marker for MPN (PV and ET) within the Ruxo-BEAT trial	Myeloproliferative neoplasms (MPN) including essential thrombocythemia (ET), polycythemia vera (PV)
**STEMVAC in Patients with Early-Stage Triple-Negative Breast Cancer**	NCT05455658	STEMVAC DNA Plasmid-Based Vaccine (STEMVAC) Encoding Th1 Selective Epitopes from Five Antigens Associated with Breast Cancer Stem Cells (MDM2, YBX1, SOX2, CDH3, CD105)	Phase 2	To test the immune system’s response to STEMVAC	Patients with stage IB-III triple-negative breast cancer
**A Multiple Antigen Vaccine (STEMVAC) for the Treatment of Patients with Stage IV Non-Squamous Non-Small-Cell Lung Cancer**	NCT05242965	STEMVAC	Phase 2	This phase II trial tests whether STEMVAC polyepitope plasmid DNA vaccine works to shrink tumors	Lung Non-Squamous Non-Small-Cell Carcinoma Stage IV Lung Cancer AJCC v8
**Vaccine Therapy in Treating Patients with HER2-Negative Stage III-IV Breast Cancer**	NCT02157051	STEMVAC	Phase 1	To study the side effects and best dose of multiantigen deoxyribonucleic acid (DNA) plasmid-based vaccine STEMVAC in treating patients with HER2-negative stage III-IV breast cancer	HER2-negative stage III-IV breast cancer

**Table 3:** Potential compounds targeting YBX1.

Name	Cancer Type	Mechanism	Regimen	Animal Study	Reference
**Azopodo-phyllotoxin SU056**	Ovarian cancer	YBX1 inhibitor	Alone 20 mg/kg daily or 10 mg/kg daily + palitaxel 5 mg/kg weekly	Xenograft	[116]
**Fisetin**	Prostate cancer Melanoma	Bind to YBX1 and hinder YBX1 from interacting with AKT Inhibit RSK kinase activity Inhibit EMT and MDR1	1mg Fisetin/animal intraperitoneally compared to 2 mg Vemurafenib/animal orally	Xenograft	[269,270]
**2,4-dihydroxy-5-pyrimidinyl imidothiocarbomate (DPI)**	Breast cancer	Inhibit YBX1 nuclear translocation	110.1 μM	Chick chorioallantoic membrane (CAM) xenograft	[272]
**7-hydroxyindirubin**	Hepatocellular carcinoma	Inhibit Actinomycin D-induced YBX1 nuclear translocation	7-hydroxyindirubin 5 μM Actinomycin D 65 nM	No	[274]
**Sesquiterpene lactone 6-O-angeloylplenolin**	Colon carcinoma HCT-8	Reverse Vincristine resistance by inhibiting YBX1 nuclear translocation	3.5–7 mg/kg/day orally Vincristine 2.5 mg/kg/day orally	Xenograft	[273]
**TAS0612**	Triple-negative breast cancer	Suppress YBX1 expression by inhibiting multikinase (AKT, p70S6K, and p90RSK)	50 mg/kg	Xenograft	[32]
**BEZ235**	Colorectal cancer	Suppress YBX1 expression through inhibiting AKT/mTOr, thus enhancing the cytotoxicity of radiotherapy	50 mg/kg once daily	Intrahepatic tumorigenesis	[275]
**Aloe-emodin**	HER2-positive breast cancer	Suppress YBX1 expression by inhibiting Twist and ILK/ATK/mTor signaling	12.5–50 mg/kg	Xenograft	[276]

## Data Availability

Not applicable.

## Abbreviations

YBX1: Y-box binding protein 1; GSK3B: glycogen synthase kinase 3 beta; EGFR: epidermal growth factor receptor; p90RSK: Ribosomal Protein S6 Kinase A1; CDC25a: Cell division cycle 25A; CCL5: C-C Motif Chemokine Ligand 5; Vegfb: Vascular Endothelial Growth Factor B; SASP: senescence-associated secretory phenotype, EMT: Epithelial-mesenchymal transition; TGFβ1: Transforming Growth Factor Beta 1; HIF1α: Hypoxia Inducible Factor 1 Subunit Alpha; MSH5: MutS Homolog 5; GOLPH3: Golgi phosphoprotein 3; lncRNA: long non-coding RNA; 5mC: methylation of cytosine at the fifth position; GBM: Glioblastoma Multiforme; SLE: Systemic Lupus Erythematosus; RIP: RNA Immunoprecipitation.
